# A novel technique for lymphadenectomy along the left recurrent laryngeal nerve during minimally invasive esophagectomy: a retrospective cohort study

**DOI:** 10.1186/s12893-023-02263-5

**Published:** 2023-11-21

**Authors:** Guo Dongming, Jiang Yuequan, Zhang Qi, Xing Huajie, Wang Zhiqiang

**Affiliations:** Department of Thoracic Cancer Center, Chongqing University Cancer Hospital, Chongqing, 400030 China

**Keywords:** Complications, Elastic suspension, Left recurrent laryngeal nerve, Lymphadenectomy, Overall survival

## Abstract

**Background:**

In the context of esophageal cancers, lymph nodes located along the left recurrent laryngeal nerve (RLN) exhibit significant involvement, posing significant challenges for lymphadenectomy. The objective of this study is to assess the safety and efficacy of a novel technique for lymphadenectomy called "elastic suspension of left RLN" method, comparing it with the conventional approach.

**Methods:**

Between January 2016 and June 2020, a total of 393 patients who underwent minimally invasive esophagectomy with gastroplasty and cervical esophagogastric anastomosis were enrolled in the study. Among them, 291 patients underwent the "elastic suspension of left RLN" method, while 102 patients underwent the conventional method. We compared the number of harvested lymph nodes along the left RLN and assessed postoperative complications between these two groups. Additionally, the overall survival (OS) rate was calculated and analyzed for the entire cohort.

**Results:**

In comparison to the conventional group, the elastic suspension group exhibited a higher yield of harvested lymph nodes along the left RLN (5.36 vs 3.07, *P* < 0.001). Moreover, the incidence of postoperative hoarseness was lower in the elastic suspension group (10.65% vs 18.63%, *P* = 0.038). The average duration of lymphadenectomy along the left RLN was 11.85 min in the elastic suspension group and 11.51 min in the conventional group, although this difference was not statistically significant (*P* = 0.091). Notably, the overall 5-year OS was markedly higher in the elastic suspension group compared to the conventional group (64.1% vs. 50.1%, *P* = 0.020).

**Conclusions:**

The findings suggest that the novel "elastic suspension of left RLN" method for lymphadenectomy along the left RLN in minimally invasive esophagectomy is both safe and effective. This technique holds promise for widespread adoption in esophagectomy procedures.

## Background

Esophageal cancer is one of the most prevalent malignant tumors worldwide, with a high morbidity and mortality rate in Asia [1, 2]. Poor prognosis primarily stems from lymph node metastases, notably in proximity to the recurrent laryngeal nerves (RLN). Notably, patients with thoracic esophageal cancer exhibit the highest incidence of RLN lymph node metastasis [3]. Studies conducted by Shiozaki et al. reported a substantial 49.1% rate of RLN lymph node metastasis [4], while Zhang et al. documented a metastasis rate of 28% along the left RLN [5]. Consequently, undertaking radical lymphadenectomy along the left RLN emerges as a crucial intervention. This procedure not only enhances postoperative survival but also ensures precise nodal staging, thereby providing invaluable guidance for postoperative treatments.

The total two-field lymphadenectomy, performed concomitantly with esophagectomy using the Mckeown procedure, has gained widespread acceptance among surgeons for the treatment of patients diagnosed with thoracic esophageal squamous cell carcinoma [6–8]. Nevertheless, executing lymphadenectomy along the RLN poses considerable challenges. These challenges arise from several limitations, including the heightened sensitivity of nerves due to thermal damage, intricate anatomical structures within the superior mediastinum, and the restricted working space available in the superior mediastinal area [9]. In comparison to the right RLN, the left RLN proves to be more challenging to expose and is susceptible to injuries during lymphadenectomy due to its deep-seated location and extended course. Studies by Koterazawa et al. and Oshikiri et al. indicated significant rates of RLN injury, with 26% and 28% incidences, respectively, during minimally invasive esophagectomy [10, 11]. Scholars have experimented with a limited array of techniques for lymphadenectomy along the left RLN in the past [11, 12]. Despite these efforts, there still lacks a universally accepted standard method for conducting lymphadenectomy along the left RLN.

We implemented a novel technique called “elastic suspension of left RLN (ESLR)” for lymphadenectomy along the left RLN with the aim of reducing nerve injury rates and enhancing lymph node dissection rates. In this study, we delineated the surgical procedure and conducted a retrospective analysis to evaluate the safety and efficacy of this technique. This evaluation involved comparing the outcomes of the ESLR method with those of the conventional method for lymphadenectomy.

## Patients and methods

### Study design and protocol

This retrospective cohort study was conducted at a single center, utilizing records from our institution. Ethical approval for this study was obtained from the Ethics Committee of our institution, and informed consent for the scientific use of medical data was obtained from all patients who were treated. The study has been retrospectively registered on Researchregistry.com.

### Participant selection

#### Setting

Consecutive patients who were diagnosed with thoracic esophageal cancer and underwent surgery at our hospital from January 2016 to June 2020, were included. A total of 393 patients were finally included in this study.

#### Eligibility criteria

i. Patients diagnosed with thoracic esophageal squamous carcinoma.
ii. History of planned minimal invasive esophagectomy.
iii. Clinical staging no more than cT3 or cN2.
iv. R0 resection was completed.

#### Exclusion criteria

i. History of surgery in neck or thorax.
ii. Coexistence of severe dysfunction of other organs such as severe hypertension, severe coronary heart disease, and severe diabetes.
iii. Metastasis in other organs.
iv. Coexistence of other malignant tumor.

#### Follow-up

The end point of this study was death or loss to follow-up. By the time the study was terminated, patients were followed up for 5.3–67.0 months (median follow-up time was 31.0 months).

### Outcome data, measures, and definitions

#### Patient demographics

The baseline characteristics of patients included age, gender, comorbidities, tumor location, clinical stages, and neoadjuvant treatment. The clinical stages were evaluated according to the American Joint Committee on Cancer and Union for International Cancer Control (Edition 7th). Some patients with cT3 or cN + tumors received neoadjuvant chemotherapy. The neoadjuvant chemotherapy protocol was cisplatin and paclitaxel plus fluorouracil.

#### Primary and secondary outcomes

The primary outcome included counting the lymph nodes that were dissected at each station of the esophagus using two different methods. The lymph node stations were numbered according to the Japanese Classification of Esophageal Cancers (Edition 11th) [13].

The secondary outcomes were the postoperative outcomes and the overall survival (OS). According to the Clavien–Dindo classification, pulmonary complications of grade II or above were included in this study.

I. Pulmonary complications included conditions that required sputum suction and replacement or increasing the antibiotic strength.
II. Anastomotic leakage was identified through radiography or gastroscopy.
III. Chyle leakage was identified based on the drainage fluid.
IV. RLN injury was diagnosed as postoperative hoarseness, choking when drinking, and vocal cord paralysis in laryngoscopy.

### Grouping criteria

Patients were categorized into two groups based on their surgery dates: 291 cases underwent lymphadenectomy along the left RLN using the ESLR method, while 102 cases underwent lymphadenectomy along the left RLN using the conventional method.

### Surgical procedure and postoperative treatment

#### Surgical procedure

##### General procedure

**Fig. 1:**
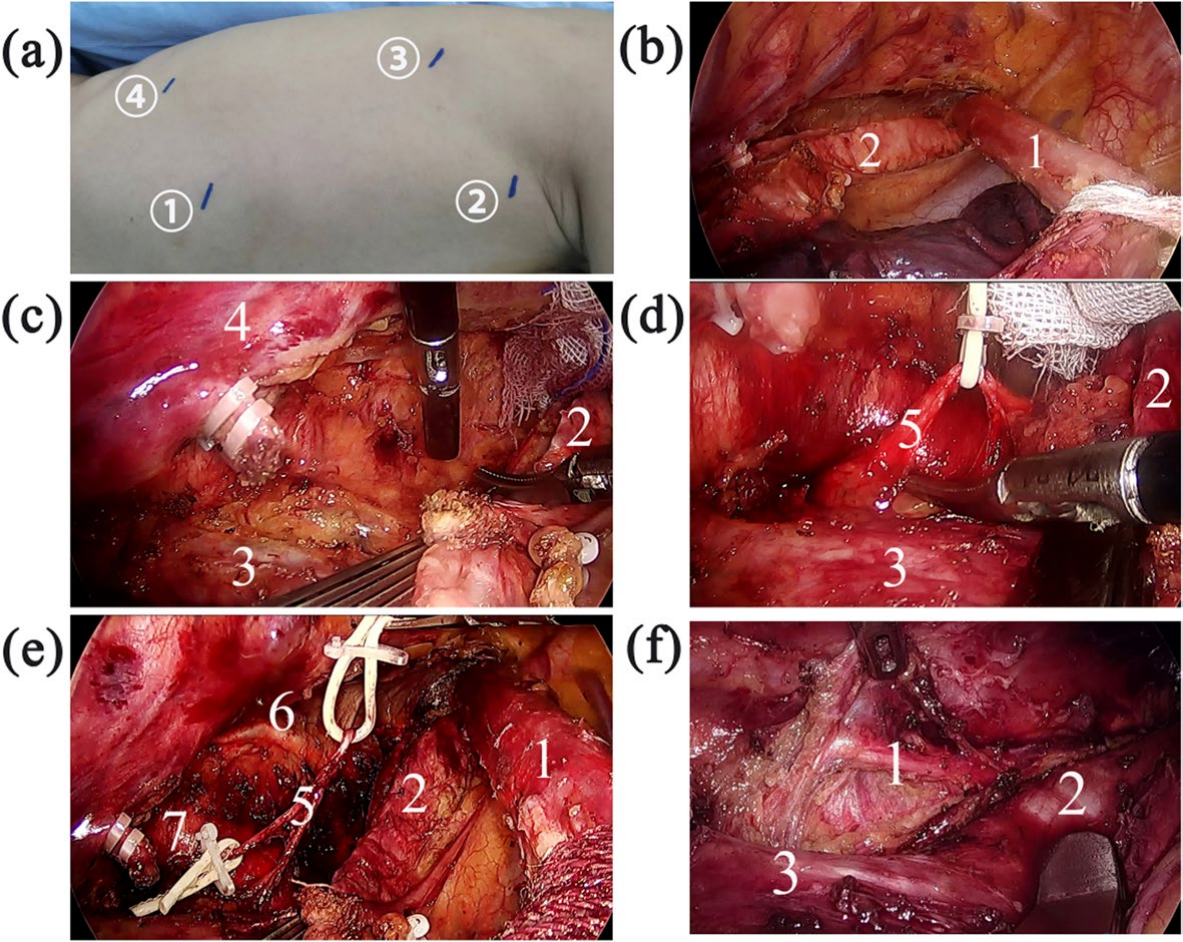
Surgical procedure*.* **a** The position of each port for thoracic surgery. **b-e** Thoracic procedure in the “elastic suspension of left RLN” method. **f** Thoracic procedure in the conventional method. *Port ① was the camera port, ② was the main operation port, ③ and ④ were the other two assistant ports. 1 = Esophagus, 2 = Trachea, 3 = Left principal bronchus, 4 = Spine, 5 = Left RLN, 6 = Thoracic duct, 7 = Aortic arch. RLN – recurrent laryngeal nerve

McKeown esophagectomy was conducted under general anesthesia with patients intubated using a single-lumen endotracheal tube. Patients were positioned in the left semi-prone posture with a 30° ventral rotation along the body's axis during the thoracic phase of the procedure. The surgeon positioned themselves on the patient's ventral side. The primary surgical port was situated at the 3rd intercostal space (ICS) along the middle axillary line. The camera port was placed at the 7th ICS along the middle axillary line. Additionally, two assistant ports were positioned: one at the 5th ICS along the subscapular line and another at the 9th ICS along the subscapular line (Fig. 1a). Following the thoracic phase, patients were repositioned horizontally for the abdominal and cervical components of the procedure, with anastomosis performed on the left side of the neck.

##### Thoracic procedure with ESLR method

Firstly, the right vagus nerve and the right RLN were mobilized, and lymphadenectomy was performed along the right RLN. The posterior mediastinal pleura was dissected along the spine from the level of the azygos arch to the top of chest. The pleura was subsequently dissected toward the right subclavian artery, along the dorsal side of the internal jugular vein. After blunt separation of the fascia with dissecting forceps, the right vagus nerve and the initial segment of the right RLN were exposed. Lymphadenectomy along the right RLN was performed subsequently. In the second phase, the thoracic esophagus was mobilized, and the arch of azygos vein was ligated and transected. The esophagus was further mobilized from the uppermost part of the chest to the diaphragmatic hiatus. During this process, the adjacent lymph nodes and mediastinal pleura were dissected. Ultimately, lymphadenectomy along the left RLN was carried out using the ESLR method.

- ① A fiber rope was threaded through the main surgical port to suspend the esophagus at the level of the azygos arch. The esophagus was drawn ventrally, revealing the trachea (Fig. 1b).
- ② The fascia in the tracheoesophageal groove was dissected along the left wall of the trachea, starting from the initial part of the left principal bronchus and extending to the uppermost part of the chest using an electric hook. Simultaneously, an assistant pushed the lower segment of the trachea and the left principal bronchus ventrally, ensuring complete exposure of the left RLN location (Fig. 1c). The left RLN was explored through blunt dissociation, and once exposed, a rubber band was placed around it for elastic suspension (Fig. 1d).
- ③ With forceps held in the left hand, the rubber band was elevated, thereby maintaining suspension of the left RLN. Utilizing the right hand, a blunt/sharp incision was made to expose the left RLN. Surrounding lymph nodes and tissues were dissected meticulously until reaching the inferior edge of the aortic arch and the base of the left pulmonary artery, ensuring a thorough dissection process.
- ④ Similar to step ③, the left RLN was completely exposed from the superior edge of the aortic arch. The surrounding lymph nodes and tissues were dissected until the cervical tissue (Fig. 1e). The inferior thyroid artery was the superior boundary of left RLN lymph node dissection.
- ⑤ From the level of aortic arch to cervical area, all lymph nodes and tissues were removed along the left RLN with an ultrasonic harmonic scalpel.

##### Thoracic procedure using conventional method

The process of esophageal mobilization and the dissection of mediastinal lymph nodes, as well as lymph nodes along the right RLN, followed identical procedures to those employed in the ESLR method. Initially, the esophagus was suspended ventrally using a fiber rope through the main surgical port before dissection of lymph nodes along the left RLN. An assistant pushed the trachea ventrally, creating optimal exposure. Subsequently, the surgeon grasped lymph nodes and surrounding tissues along the left RLN with the left hand, utilizing forceps. Due to gravity, the left RLN naturally descended. Using an ultrasonic harmonic scalpel, the surgeon dissected lymph nodes along the left RLN with the right hand (Fig. 1f).

#### Postoperative treatment

Post-surgery, patients received total parenteral nutrition and proton pump inhibitors for a duration of 7 days. Subsequently, they were transitioned to an oral liquid diet. All patients with pathological stage pT1-4N1-3M0 received standard adjuvant chemotherapy and adjuvant radiotherapy. The adjuvant chemotherapy regimen was paclitaxel and cisplatin. Regular follow-up appointments were scheduled for all patients to monitor their progress and recovery.

#### Statistical analysis

The date was analyzed using SPSS 22.0 software. The mean values were compared using the Student’s *t*-test. Differences between proportions were compared using the chi-squared test. OS was analyzed using the Kaplan–Meier methodology and Cox proportional hazard regression model. Univariate and multivariate analyses were used in the Cox regression model. A *P* value less than 0.05 was considered statistically significant.

## Results

### Patient characteristics and outcomes

#### Patient characteristics

The study comprised 393 patients, with 291 in the "ESLR group" and 102 in the "conventional group," categorized based on the lymphadenectomy method employed along the left RLN. The operation was performed by two surgeons separately, who had independently completed more than 200 cases of minimally invasive esophagectomy before this study and had rich and stable surgical experience. All the patients were Chinese. The average age of all participants was 60.51 years. There were no significant differences in age, sex, tumor location, clinical stages, or the number of patients operated under different surgeons between the ESLR and conventional groups. (Table 1).

**Table 1:** Preoperative characteristics of patients

	ESLR group *(n* = 291)	Conventional group (*n* = 102)	*P*
Age (years)	60.79 ± 7.96	59.72 ± 8.80	0.253
Gender (n)			0.211
male	241(82.82%)	90(88.24%)	
female	50(17.18%)	12(11.76%)	
Comorbidity (n)			
Hypertension	105(36.08%)	34(33.33%)	0.633
Diabetes	41(14.09%)	12(11.76%)	0.617
COPD	32(11.00%)	15(14.71%)	0.375
Others	21(7.22%)	9(8.82%)	0.665
Tumour location^a^ (n)			0.728
Upper thoracic	21(7.22%)	5(4.91%)	
Middle thoracic	162(55.67%)	59(57.84%)	
Lower thoracic	108(37.11%)	38(37.25%)	
Clinical stages (n)			0.888
I	43(14.78%)	13(12.75%)	
II	187(64.26%)	67(65.69%)	
III	61(20.96%)	22(21.56%)	
Neoadjuvant treatment (n)	97(33.33%)	30(29.41%)	0.274
Surgeons (n)			0.222
Doctor 1	105(36.08%)	30(29.41%)	
Doctor 2	186(63.92%)	72(70.59%)	

*ESLR* Elastic suspension of left recurrent laryngeal nerve, *COPD* Chronic obstructive pulmonary disease. ^a^ *Upper thoracic* From the level of superior aperture of thorax to the level of inferior edge of the azygos arch. *Middle thoracic* From the level of the inferior edge of the azygos arch to the level of the inferior edge of the inferior pulmonary vein. *Lower thoracic* From the level of the inferior edge of the inferior pulmonary vein to the esophagogastric junction

#### Surgical outcomes of the patients

All patients underwent successful surgeries without the requirement of thoracotomy. The average duration of lymphadenectomy along the left RLN was 11.85 min in the ESLR group and 11.51 min in the conventional group, with no significant difference noted (*P* = 0.091). Additionally, there were no significant differences observed in tumor size, pathologic T stage, or N stage between the two groups. The thoracic duct was not routinely resected unless injury was detected during surgery, and there was no notable difference in the incidence of patients requiring thoracic duct resection between the groups (Table 2). Total two-field lymph node dissection was carried out for all patients. Comparison of the number of harvested lymph nodes in each lymph node station revealed no significant difference in the total number of harvested lymph nodes between the two groups. However, there was a notable disparity in the number of harvested lymph nodes along the left RLN, with the ESLR group yielding a higher count than the conventional group (5.36 vs. 3.07, *P* < 0.001) (Tables 2 and 3).

**Table 2:** Surgical outcomes of the patients

	ESLR group (*n* = 291)	Conventional group (*n* = 102)	*P*
Operative time (min)	260.40 ± 26.49	255.25 ± 27.91	0.096
Chest	61.43 ± 8.20	59.66 ± 8.51	0.064
Lymphadenectomy along left RLN	11.85 ± 2.08	11.51 ± 1.57	0.091
Total number of LNs (n)	32.71 ± 6.99	30.47 ± 6.87	0.005
Number of LNs along the left RLN (n)	5.36 ± 1.35	3.07 ± 1.09	< 0.001
Size of tumor (cm)	3.54 ± 1.20	3.60 ± 1.12	0.666
Pathologic T stage (n)			0.415
0	7(2.41%)	3(2.94%)	
1	45(15.46%)	16(15.69%)	
2	43(14.78%)	22(21.57%)	
3	196(67.35%)	61(59.80%)	
Pathologic N stage (n)			0.426
0	139(47.77%)	58(56.86%)	
1	90(30.93%)	28(27.45%)	
2	45(15.46%)	12(11.77%)	
3	17(5.84%)	4(3.92%)	
Thoracic duct resection (n)			0.957
Yes	28(9.62%)	10(9.80%)	
N0	263(90.38%)	92(90.20%)	

*ESLR* Elastic suspension of left recurrent laryngeal nerve, *RLN* Recurrent laryngeal nerves, *LNs* Lymph nodes

**Table 3:** Number of harvested lymph nodes and postoperative complications of the patients

	ESLR group (n = 291)	Conventional group (n = 102)	*P*
Number of lymph nodes (n, 11th JES map)			
106recR	3.10 ± 1.09	3.16 ± 1.18	0.676
106recL	5.36 ± 1.35	3.07 ± 1.09	< 0.001
Middle and lower mediastinum	15.87 ± 15.26	15.13 ± 4.72	0.209
Enterocoelia	8.37 ± 3.57	9.12 ± 4.27	0.085
Postoperative complications (n)			
RLN injury	31 (10.65%)	19 (18.63%)	0.038
Pneumonia	14 (4.81%)	9 (8.82%)	0.137
Anastomotic leakage	17 (5.82%)	7 (6.86%)	0.711
Chyle leakage	6 (2.05%)	4 (3.92%)	0.305
Others	6 (2.05%)	6 (5.88%)	0.054

*ESLR* Elastic suspension of left recurrent laryngeal nerve, *JES* Japanese Esophageal Society, *RLN* Recurrent laryngeal nerves

The occurrence of postoperative hoarseness was significantly lower in the ESLR group compared to the conventional group (31 cases, 10.65% vs. 19 cases, 18.63%, *P* = 0.038). Among the patients who experienced hoarseness in the ESLR group, 3 individuals showed partial recovery two months after surgery. Additionally, both groups had patients who faced postoperative pneumonia; however, these cases (14 in the ESLR group and 9 in the conventional group) successfully recovered following antibiotic treatment. Moreover, anastomotic leakage occurred in 17 patients from the ESLR group and 7 patients from the conventional group; fortunately, all patients survived this complication (Table 3). Importantly, there were no fatalities within the initial 30 days post-surgery.

### Overall survival of patients

**Fig. 2:**
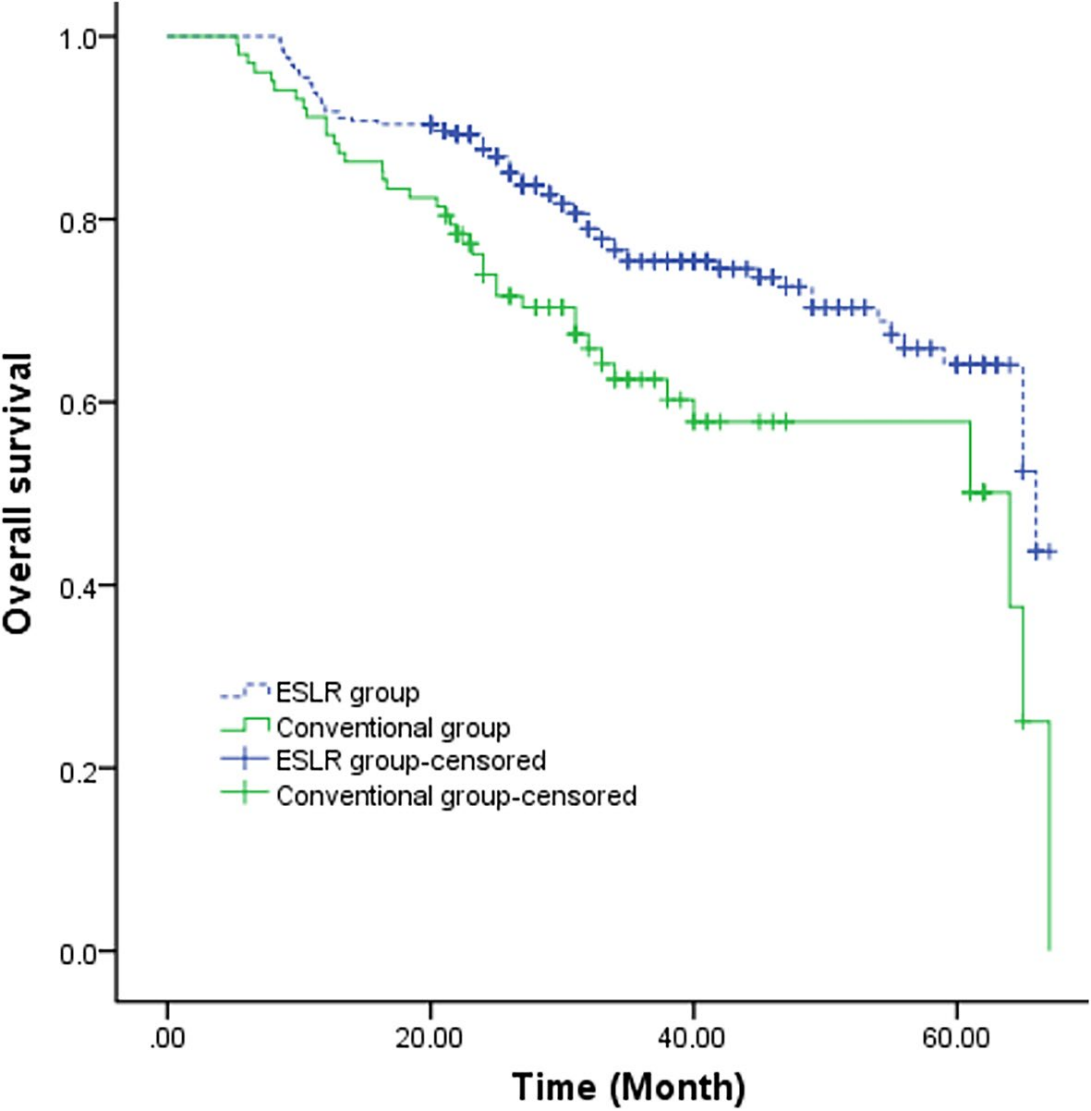
Kaplan–Meier survival curve for overall survival in the ESLR group and conventional group. ESLR – elastic suspension of left recurrent laryngeal nerve

Patients were followed up for 5.3–67.0 months (median follow-up time was 31.0 months). By the conclusion of the follow-up period, 10 patients had been lost to follow-up, resulting in an overall follow-up rate of 97.46%. Notably, the 5-year OS rate was markedly higher in the ESLR group compared to the conventional group (64.1% vs. 50.1%, *P* = 0.020), (Fig. 2).

**Fig. 3:**
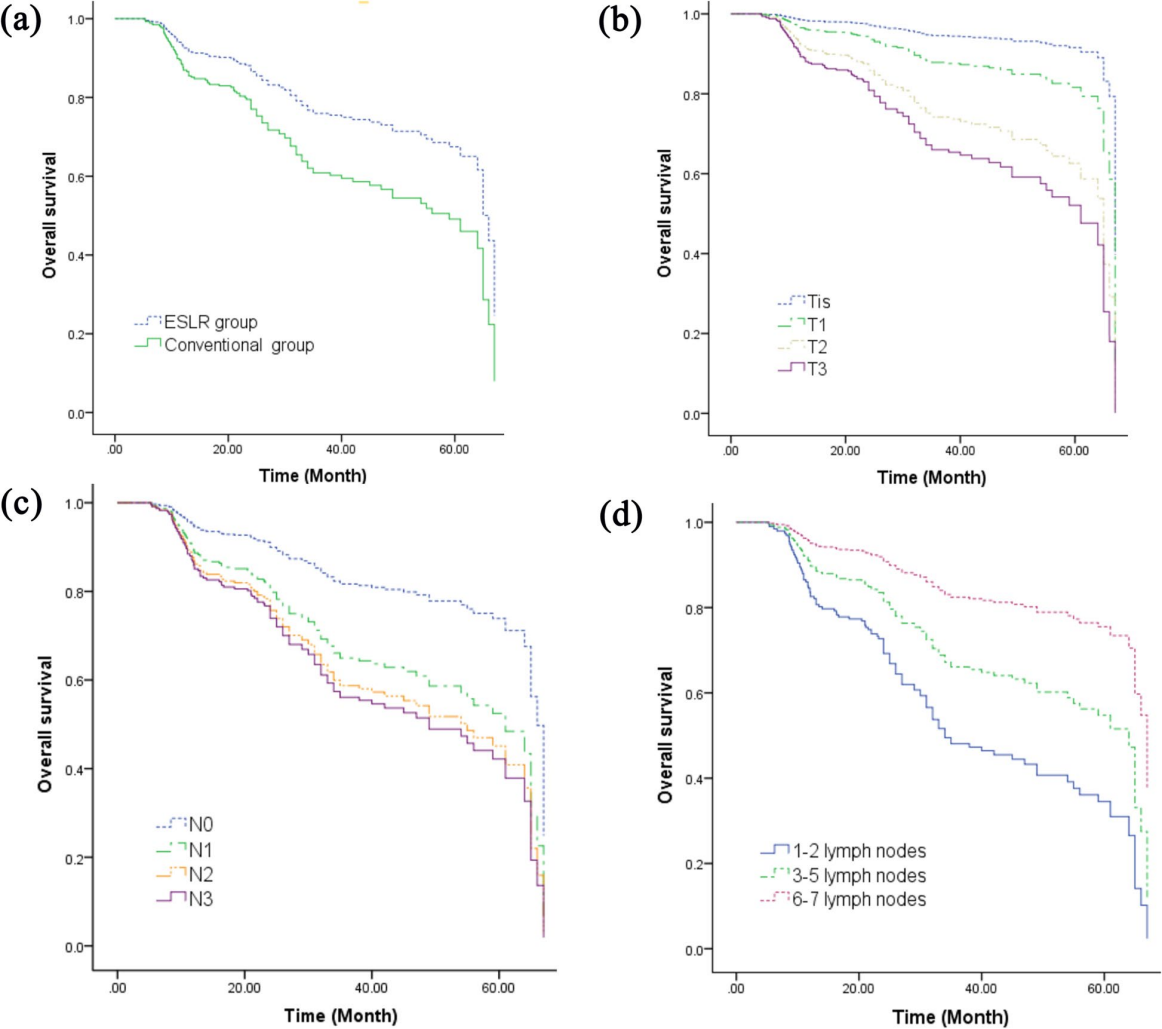
Curves for overall survival (OS). **a** OS for different types of surgery. **b** OS for different pathologic T stages. **c** OS for different pathologic N stages. **d** OS for different number of harvested lymph nodes along the left recurrent laryngeal nerve. ESLR – elastic suspension of left recurrent laryngeal nerve

The significant factors, influencing OS, as identified through both univariate and multivariate analyses, are outlined in Table 4. OS was found to be influenced by the type of surgery, pathological T (pT) and N (pN) stages, as well as the number of harvested lymph nodes along the left RLN, as indicated in Table 4 and illustrated in Fig. 3.

**Table 4:** Univariate and multivariate Cox regression analyses of overall survival

Factors	Univariate analysis		Multivariate analysis	
	Hazard ratio (95% CI)	*P*	Hazard ratio (95% CI)	*P*
Type of surgery		0.002		< 0.001
ESLR method	0.744 (0.614–0.902)		0.409 (0.274–0.612)	
Conventional method	1(reference)		1(reference)	
Age	1.027 (1.002–1.052)	0.034		
Total number of harvested LNs	0.973 (0.947–1.000)	0.052		
pT stage		0.001		0.019
Tis	0.135 (0.018–0.995)		0.159 (0.021–1.203)	
T1	0.312 (0.158–0.615)		0.403 (0.198–0.821)	
T2	0.718(0.433–1.191)		0.596 (0.352–1.010)	
T3	1(reference)		1(reference)	
pN stage		< 0.001		0.001
N0	0.350 (0.169–0.728)		0.474 (0.225–0.997)	
N1	0.747 (0.362–1.540)		1.023 (0.492–2.128)	
N2	0.922 (0.422–2.015)		1.237 (0.562–2.724)	
N3	1(reference)		1(reference)	
Number of harvested LNs along the left RLN		< 0.001		< 0.001
1–2	1.886 (1.359–2.618)		4.343 (2.420–7.794)	
3–5	1.066 (0.828–1.373)		2.542 (1.635–3.951)	
6–7	1(reference)		1(reference)	
Neoadjuvant treatment		0.278		
No	1.261 (0.829–1.920)			
Yes	1(reference)			

*ESLR* Elastic suspension of left recurrent laryngeal nerve, *LNs* Lymph nodes, *RLN* Recurrent laryngeal nerve

## Discussion

Zheng et al. previously introduced an esophageal suspension method aimed at reducing postoperative hoarseness following esophagectomy [9]. However, this approach did not demonstrate any advantages concerning the number of harvested lymph nodes along the recurrent laryngeal nerves (RLNs) when compared to the non-suspension group, as observed in prior studies [14–16]. Additionally, Oshikiri et al. employed the "Bascule Method" for lymph node dissection along the left RLN during esophagectomy [11]. Despite its efficacy, the prone position utilized in this method posed challenges in emergency situations, such as sudden massive bleeding or when urgent conversion to thoracotomy was necessary [17]. Furthermore, the Bascule Method is unsuitable for patients with tumors located across the aortic arch. In the pursuit of minimizing RLN injury, Otsuka et al. placed greater impetus on micro-anatomical layers [18]. However, this approach demands additional time during lymphadenectomy compared to the conventional method. Moreover, the procedure involves two distinct parts, the thoracic and cervical procedures, to accomplish the lymphadenectomy process. In our efforts to achieve radical lymph node dissection along the left RLN while safeguarding its integrity, we introduced the innovative ESLR method. This approach not only enhanced the number of dissected lymph nodes along the left RLN but also significantly reduced the incidence of postoperative RLN injury, all without prolonging the duration of surgery. Importantly, this novel method proved to be versatile, applicable to tumors located at various positions within the thoracic region. Moreover, in comparison to alternative techniques, our novel method was found to be more accessible and easier to master.

Esophageal cancer is predominantly prevalent in Asia and East Africa. In the progression of esophageal cancer, lymph node metastasis occurs early and is frequently characterized as skips or bi-directional metastasis [3, 19]. Lymph nodes along RLNs are highly involved. Tan et al. reported a lymph node metastasis rate along the RLN of 26%, with 20.9% occurring along the right side and 8.7% along the left side [20]. Tachimori et al. reported the incidence of RLN lymph node metastasis as 13.6% along the left side [21]. Radical lymphadenectomy along RLNs could improve the prognosis [22, 23]. In this study, the implementation of the novel ESLR method led to a higher number of dissected lymph nodes along the left RLN compared to the conventional method. Furthermore, patients in the ESLR group exhibited a superior 5-year OS rate in comparison to patients in the conventional group. In the multivariate Cox regression analysis, the number of harvested lymph nodes along the left RLN emerged as a significant factor associated with OS. Specifically, the removal of a greater number of lymph nodes along the left RLN correlated with a more favorable prognosis in this study. These results are consistent with findings from previous studies [22, 23].

Achieving adequate operative exposure is the first step in the lymphadenectomy along RLNs. Compared to the right RLN, the left RLN was more difficult to expose and mobilize through the right thorax. The left RLN resides within a confined and deep space, and its trajectory is longer compared to the right RLN [24]. When patients assumed the left lateral position, the prolapsed right lung and mediastinal structures and organs naturally shifted toward the left side, further deepening the location of the left RLN. During the chest procedure in the ESLR method, patients were positioned in the left semi-prone posture with a 30° ventral rotation of the body axis. In this specific posture, the right lung descended toward the ventral side, enhancing the visibility and accessibility of the operative field. This novel method involved the use of single lumen endotracheal intubation and artificial pneumothorax for all patients. The single lumen endotracheal tube, characterized by its smaller diameter and softer composition compared to a double lumen endotracheal tube, facilitated maneuverability. Unlike double lumen endotracheal intubation, the trachea could be more effectively pushed toward the ventral side by an assistant when utilizing the single lumen endotracheal intubation approach [25, 26]. Preceding the lymphadenectomy, the suspension of the esophagus and the ventral push of the trachea collectively widened the space surrounding the left RLN. The synergistic implementation of these techniques in this novel method significantly enhanced the operative exposure along the left RLN, thereby facilitating the lymphadenectomy procedure. Furthermore, following elastic suspension of the left RLN, the path of the left RLN became distinctly visible compared to the conventional method. This enhanced clarity allowed for a more meticulous and radical dissection of the lymph nodes along the left RLN.

In the postoperative phase, RLN injury stands out as a significant complication [27, 28]. This injury leads to incomplete closure of the vocal cord folds, limiting patients' ability to cough effectively. Moreover, RLN injury heightens the risk of pulmonary complications due to an increased likelihood of aspiration pneumonia. Beyond hoarseness, RLN injury can cause symptoms such as stridor, aspiration pneumonia, severe cough, and even apnea, severely impairing patients' overall quality of life. Oshikiri et al. reported an incidence of 28% for left RLN injury when employing the "Bascule method" for lymphadenectomy [11]. In the ESLR group in this study, 31 patients experienced postoperative RLN injury. The rate of RLN injury associated with this novel method was 10.65%, which was lower than that in the conventional group. Additionally, this injury rate was also lower when compared to rates reported in other studies [10, 19, 29]. Among the patients in the ESLR group who experienced hoarseness, three individuals demonstrated partial recovery within two months following surgery. It is noteworthy that the nerve tissue is highly susceptible to stretching damage [18, 30]. In our approach, we utilized an elastic rope to suspend the left RLN. The effect of elastic traction exerted by the rope on the left RLN mimicked the physiological tension from the surrounding peripheral tissues, possibly enhancing safety during the procedure. Notably, nerves are highly sensitive to clamping and thermal damage [9, 30]. Following the suspension of the left RLN, it was simultaneously "marked" using an elastic rope, providing a distinct visual indicator for surgeons to differentiate the left RLN from the surrounding connective tissues. Employing this novel method ensured a sufficient distance between the suspended left RLN and the adjacent tissues. Consequently, the nerve remained safeguarded against thermal damage during the lymphadenectomy procedure.

It is essential to acknowledge the limitations of this study. The retrospective nature of the study design imposed constraints on randomization, leading to potential bias in the selection of surgical techniques. Moreover, our study solely focused on OS, without delving into disease-free survival rates and tumor-related deaths. To address these limitations, future studies should consider adopting a prospective and randomized approach to provide more robust and comprehensive insights into the effectiveness of the surgical techniques employed.

## Conclusions

The utilization of the ESLR method in lymph node dissection along the left RLN during minimally invasive esophagectomy has been demonstrated to be both safe and effective. This innovative approach not only enhances the quantity of dissected lymph nodes along the left RLN but also significantly reduces the incidence of postoperative RLN injury, all without causing a prolongation of operative time. Moreover, patients subjected to this method may experience improved long-term survival rates. Considering these advantages, the ESLR method holds the potential for widespread adoption in esophagectomy procedures.

## Data Availability

The datasets used and analyzed during the current study are available from the corresponding author on reasonable request.

## Abbreviations

RLN: Recurrent laryngeal nerve
ESLR: Elastic suspension of left recurrent laryngeal nerve
OS: Overall survival
ICS: Intercostal space
COPD: Chronic obstructive pulmonary disease
LNs: Lymph nodes
JES: Japanese esophageal society
